# Eating Disorders across the life span: the role of biology and psychosocial factors

**DOI:** 10.1192/j.eurpsy.2023.87

**Published:** 2023-07-19

**Authors:** N. Micali

**Affiliations:** Mental Health Services of the Capital region of Denmark, Copenhagen, Denmark

## Abstract

**Abstract:**

Eating Disorders are common. They onset in adolescence and affect individuals of all ages. Women are more affected than men. I will present evidence on the epidemiology of eating disorders across the lifespan. I will also review risk factors focusing in particular on biological risk factors that might explain onset of these disorders in critical periods of a woman’s life. I will also cover pyschosocial risk factors across life stages. I will present results from a series of cohort studies. I will then summarise the evidence from our own and other existing studies.

**Disclosure of Interest:**

None Declared

